# Preclinical and clinical advances to overcome hypoxia in glioblastoma multiforme

**DOI:** 10.1038/s41419-024-06904-2

**Published:** 2024-07-13

**Authors:** Jolie Bou-Gharios, Georges Noël, Hélène Burckel

**Affiliations:** 1 Institut de Cancérologie Strasbourg Europe (ICANS), Radiobiology Laboratory, 3 rue de la porte de l’Hôpital, 67000 Strasbourg, France; 2 Laboratory of Engineering, Informatics and Imaging (ICube), Integrative Multimodal Imaging In Healthcare (IMIS), UMR 7357, University of Strasbourg, 4 rue Kirschleger, 67000 Strasbourg, France; 3 Institut de Cancérologie Strasbourg Europe (ICANS), UNICANCER, Department of Radiation Oncology, 17 rue Albert Calmette, 67200 Strasbourg, France

**Keywords:** CNS cancer, CNS cancer

## Abstract

Glioblastoma multiforme (GBM) is the most common adult primary brain tumor. The standard clinical treatment of GBM includes a maximal surgical resection followed by concomitant radiotherapy (RT) and chemotherapy sessions with Temozolomide (TMZ) in addition to adjuvant TMZ cycles. Despite the severity of this protocol, GBM is highly resistant and recurs in almost all cases while the protocol remains unchanged since 2005. Limited-diffusion or chronic hypoxia has been identified as one of the major key players driving this aggressive phenotype. The presence of hypoxia within the tumor bulk contributes to the activation of hypoxia signaling pathway mediated by the hypoxia-inducing factors (HIFs), which in turn activate biological mechanisms to ensure the adaptation and survival of GBM under limited oxygen and nutrient supply. Activated downstream pathways are involved in maintaining stem cell-like phenotype, inducing mesenchymal shift, invasion, and migration, altering the cellular and oxygen metabolism, and increasing angiogenesis, autophagy, and immunosuppression. Therefore, in this review will discuss the recent preclinical and clinical approaches that aim at targeting tumor hypoxia to enhance the response of GBM to conventional therapies along with their results and limitations upon clinical translation.

## Facts

- Tumor hypoxia is linked to the aggressive nature of Glioblastoma Multiforme (GBM) leading to first-line therapy resistance and recurrence.
- Hypoxia is still overlooked in the clinical management of GBM.
- Preclinical and clinical strategies to combat tumor hypoxia in GBM and enhance its response to treatments and their limitations.
- Potential integration of tumor hypoxia in the diagnosis and treatment of GBM could lead to a different disease management and outcome.

## Open questions

- Based on the role of tumor hypoxia in GBM, would targeting hypoxia enhance the response of GBM to first-line therapies?
- How can the proposed preclinical and clinical strategies targeting hypoxia influence the outcome of GBM?
- What are the limitations of the proposed hypoxia-targeting approaches on their clinical translation?

## Introduction

The first-line therapy for treating newly diagnosed GBM was based on the phase III clinical trial carried out by the European Organization for Research and Treatment of Cancer (EORTC), and the National Cancer Institute of Canada Clinical Trials Group (NCIC CTG) [1, 2]. The possibility of a maximal surgical resection is determined, followed by concomitant radiotherapy (RT) and chemotherapy, namely Temozolomide (TMZ). Although the median overall survival (OS) of GBM patients ranges between 12 and 18 months and the relative 5-year survival rate since diagnosis is less than 7% regardless of the gender, yet the conventional clinical protocol of GBM remains unchanged since 2005, except for the approval of the tumor-treating field (TTF) [3]. Clinical studies showed that the addition of TTF to chemotherapy (TMZ) post-chemoradiotherapy, prolonged the median PFS to 6.7 months versus 4 months for TMZ group and revealed a significant effect of TTF on PFS and OS [4, 5].

Over the years, some innovative technologies have been implicated to ameliorate the OS of patients, and to provide a better tumor control and enhance the quality of life (QoL) of patients. For instance, advances in RT delivery techniques offered higher precision in targeting tumors, better dose deposit, less dose diffusion in organs at risk, and therefore improved tumor control while limiting side effects and toxicities whenever possible. In parallel, extensive research on GBM has been directed towards the discovery of novel therapeutic strategies such as CAR-T cell therapy, gold or gadolinium nanoparticles, and vaccines among others, to overcome the limitations of the current standard of care treatments. Unfortunately, hypoxia is still overlooked when it comes to GBM treatment strategies despite its tremendous repercussions on GBM aggressiveness and resistance to the available treatment options. Hence, in the first part of the following section, we will describe different preclinical experiments that target hypoxia in GBM. Similarly, in the second part, we will review some clinical approaches that have assessed the efficacy of certain techniques or molecules on targeting hypoxia, through either elevating oxygen levels and diffusion, inhibition of hypoxia-related activated pathways, or via targeting angiogenesis in the context of GBM treatment.

## Preclinical approaches targeting hypoxia in GBM

### Inhibition of the hypoxia transcription factors HIF-1α and HIF-2α

Due to the major implication of hypoxia in the increased malignancy of GBM and its poor prognosis, some studies have focused on targeting the key transcription factors activating the hypoxia-signaling pathway. Therefore, several inhibitors of HIF-1α and HIF-2α have been synthesized over the years to counteract the impact of hypoxia on several diseases. Inhibitors of HIF-1α and HIF-2α are currently used in preclinical studies and numerous clinical trials as potential treatments for clinical pathologies, including but not limited to leukemia, renal cell, hepatocellular, and colorectal carcinomas. Concerning GBM, only few HIF-α inhibitors have been investigated due to the complexity of this cancer and its heterogeneous nature, in addition to its location, which renders the accessibility via the circulatory system challenging.

The high number of HIF-1α inhibitors reflects the extensive research performed in several fields including cancer, diabetes, ischemic and cardiovascular diseases, stroke, and inflammation [6]. The mode of action of HIF-1α inhibitors varies among these agents, where some inhibitors interfere at the mRNA level to block the transcription and expression of HIF-1α, such as OKN-007, while others inhibit the translation of HIF-1α mRNA, like EZN-2208. Other inhibitors, including Melatonin, Curcumin, and EF-24, promote HIF-1α degradation. Finally, some inhibitors impede the binding of HIFs to their HRE domain, such as Acriflavine, Echinomycin, and KCN1 [6, 7]. The level of inhibition, mode of action, and results from studies on HIF-1α inhibitors tested in GBM are summarized in Table 1.

**Table 1:** Pharmacological inhibition of HIF-1α and HIF-2α in GBM.

Transcription factor	Level of inhibition	Molecule	Mode of action	Studies results
HIF-1α	mRNA	OKN-007	Downregulates HIF-1 mRNA	- Opened the BBB for few hours in a mouse model [89] - Reduced the expression of HIF-1, GLUT-1, and VEGFR2 in mice models under hypoxic conditions [7] - Sensitized TMZ-resistant GBM, decreased tumor volume, and normalized tumor blood vasculature in a rat model [90]
	Protein	103D5R	Inhibits HIF-1 translation	*-* Downregulated VEGF and Glut-1 in GBM, breast, and prostate cell lines [91]
		KC7F2	Inhibits HIF-1 translation	*-* Decreased the effective dose of TMZ and effectively reduced the aerobic glycolysis of U-87 MG cell line in vitro [92]
		Cardiac glycosides (Proscillardin A, Gamabufotalin, Anvirzel™)	Inhibit the HIF-1 protein but do not downregulate its expression	- Proscillardin A reduced migration in GBM via the activation of GSK-3β and the phosphorylation of EB1 and the alteration of microtubule dynamics (U-87 MG, U-251 MG, and GBM stem-like cells GBM6, GBM9) [93] - Gamabufotalin increased GBM sensitivity to TMZ and contributed to the overphosphorylation of p38 through ATP1A3 and AQP4 (U87, U251, LN229, LN18, A172 and T98 cells) [94] - Anvirzel™ reduced GBM growth in vitro and decreased the expression of GSK-3β and HIF-1α (U-87 MG) [95]
		PX-478	Inhibits HIF-1 deubiquitination	- Downregulated VEGF expression - Decreased PD-L1 expression under hypoxia (U-251 MG, U343, GL-261) [96]
		Vorinostat	Inhibits HIF-1α nuclear translocation via acetylation of associated chaperon Hsp90	- Specific inhibition of HDAC6, decreased EB1 expression, and suppressed microtubule instability in GBM cell line (U87-MG, GL261, and GBM6) [97] - A combination with Trichostatin-A inhibited Wnt-dependent pathways, and reduced cell proliferation of primary GBM culture [98]
		Panobinostat	Induces prolonged hyperacetylation of the histone protein (histone deacetylase inhibitor)	- Induced metabolic reprogramming by lowering ATP levels, suppressing c-Myc protein levels, and elevating PGC1α and PPARD, involved in oxidative metabolism in vitro (LN229 and U-87 MG) [99] - Upregulated the estrogen receptor ERβ 1 isoform, a tumor suppressor in GBM in vitro (U87 and U251) [100]
		Topotecane	Inhibits the transcriptional activity and accumulation of HIF	- Inhibited SUMOylation, decreased levels of CDK6 and HIF-1α, and altered the cell cycle in vitro (U251, LN229, and Mz18) [101]
		Icaritin	Suppresses de novo synthesis and promotes degradation of HIF-1	- Decreased the expression of HSP-70, HSP-60, and HIF-1α in an in vitro model of cerebral ischemia (PC12 cell line) [102] - Suppressed the IL-6/Stat pathway, reduced glycolysis and decreased cell viability of human GBM cell lines in vitro (U87 and T98G) [103] - Inhibited the migratory potential of U-87 MG in vitro via PTEN/Akt signaling pathway [104]
	Binding to HRE	Acriflavine	Blocks the binding of HIF-1 to DNA	- Inhibited tumor growth in vivo in orthotopic glioma models (rodents) via intratumoral administration with biodegradable polymeric wafers (U87, GL2361, F98, 9 L gliosarcoma, GB1A (0913), and JHH1113) [105] - Suppressed tumor growth in vivo when combined to photodynamic therapy (U251 and GL261) [106]
		Echinomycin	Blocks the binding of HIF-1 to DNA	- Induced apoptosis, inhibited tumor growth in vivo, and reduced the activation of AKT pathway through PDGF-D and PDGFRα axis inhibition (U251, U87MG, GL261, and primary GBM) [107]
		KCN1	Inhibits HIF-1 transcriptional activity	- Interfered in the interaction between HIF-1 and its coactivators p300/CBP (U251MG, D54MG, D645MG, and LN443) and penetrated the BBB in a mouse GBM model (LN229) [108]
HIF-2α	Protein binding	PT2385	Blocks allosterically the dimerization of HIF-2α with HIF-β	- PT2385 alone reduced the OS of an in vivo mouse model (patient-derived GBM), however, no increase was detected in OS after combining PT2385 to conventional treatments (RT and TMZ) [8]

*HIF* hypoxia induced factor, *OKN-007* *OKlahoma Nitrone 007,* *GLUT-1* Glucose transporter 1, *VEGF* vascular endothelial growth factor, *VEGFR* vascular endothelial growth factor receptor, *HDAC6* histone deacetylase 6, *EB1* End-binding 1, *PGC1α* Pparg coactivator 1 alpha, *PPARD* Peroxisome proliferator-activated receptor delta, *ERβ1* estrogen receptor beta 1, *CDK6* cyclin-dependent kinase 6, *HSP* Heat shock protein

Although HIF-1α represents a major player and a potential target of hypoxia in cancer, its inhibition raises some concerns of unintentional side effects linked to its ubiquitous expression pattern especially in non-tumoral sites. Therefore, the therapeutic specificity of HIF-1α inhibition is debatable. On the other hand, recent studies claim that the expression of HIF-2α is more specific to tumor tissues rather than normal ones. One study proved that this is in accordance with the REMBRANDT glioma database (834 patients) which states that a poorer patient outcome and a higher glioma grade were correlated with a higher expression of HIF-2α in tumor tissue [8]. Despite the role that HIF-2α plays in GBM malignancy, only few selective inhibitors to target HIF-2α exist, and are designed to bind selectively the PAS-B domain and block its allosteric heterodimerization with HIF-1β in the nucleus [9]. So far, only one preclinical study has described the effect of PT2385 alone or in combination with RT and TMZ on GBM patient-derived cell lines. Notably, in the mentioned preclinical study, the median OS of mice in the single-arm group, treated with PT2385 only, was higher than that of untreated mice. However, the combination of PT2385 with the conventional protocol of TMZ and RT did not increase the OS of treated mice as compared to those treated with the combination [8] (Table 1). Further studies focusing on the inhibition of HIF-2α in GBM should be performed due to the association of HIF-2α with an increased mortality rate among GBM patients, with caution to the selection of GBM patients based on the expression profile of HIF-2α, to better sort patients that might benefit from this inhibition.

### Reoxygenation

#### Crocetin and trans-sodium crocetinate

Another possible approach to reverse tumor hypoxia is to modulate the oxygen concentration delivery into the hypoxic regions. For instance, trans-sodium crocetinate or TSC (C_20_H_22_Na_2_O_4_) is a trans-isomer salt compound derived from crocetin (C_20_H_24_O_4_) [10]. A preclinical study using four GBM cell lines showed that crocetin activated cell death via apoptosis, reduced cell migration, and decreased the expression of mesenchymal markers including CD44, and OCT3/4 [11]. However, some major limitations have faced the therapeutic application of crocetin due to its solubility and bioavailability issues. Therefore, TSC has been proposed as a possible solution, due to its structural configuration with a hydrophobic interior part and a polar head. TSC binds the water molecules surrounding the tissues to form a densely packed and well-organized water matrix. This increases the hydrogen bonding thus decreasing the flow resistance and reducing the density of the plasma fluid to facilitate the passage of available oxygen into hypoxic tissue sites [12]. The effect of TSC on tumor reoxygenation, along with its radiosensitizing effect have been investigated in vivo on a C6 glioma rat model [13]. TSC was administered as an intravenous bolus injection over five consecutive days with low- and moderate-dose concentrations (50–100 µg/kg), and a single dose of 8 Gy irradiation was performed. Results showed that both tumor size and growth rate were significantly reduced in the combination groups (RT and TSC) versus the single-arm groups (TSC only and RT only), in addition to the increased median survival observed in the combination group [13]. However, the pharmacokinetic profile of TSC in vivo revealed that the maximum concentration was detected 30 min post-injection, therefore posing some technical concerns for its administration during patient positioning and irradiation process [14].

#### Myo-inositol trispyrophosphate

Myo-inositol trispyrophosphate hexasodium salt (ITPP) is an allosteric effector of hemoglobin, which increases the oxygen-release capacity of red blood cells (RBCs) by reducing their oxygen-binding affinity, thus causing a better oxygen release into hypoxic regions [15]. Following its synthesis, ITPP has emerged as an anti-cancer agent, with a particular interest in GBM due to its capacity to cross the BBB and its reoxygenation properties. Different tumor models, including head and neck and non-small cell lung cancer have been implicated in preclinical studies to assess the radiosensitizing effect of ITPP [16]. Concerning GBM, one preclinical study used the 9L-glioma rat model to explore the oxygenation effect of ITPP and its potential radiosensitizing property [17]. An intraperitoneal injection of ITPP was administered at 2 g /kg once for 2 days combined with a single dose of 30 Gy delivered 2 h after the second ITPP injection. Results were highly heterogeneous, where some rats were completely cured while others showed similar tumor growth as the RT-only group [17]. Conversely, another preclinical study in a rat GBM model demonstrated that no additional effect has been observed in the ITPP treatment group [18]. Similarly, another study using the GL-261 mouse GBM model showed that the combination of ITPP with hypofractionated radiation reduced the effectiveness of RT alone [19]. Interestingly, ITPP recognizes and activates endothelial PTEN, which participates in normalizing tumor vessels instead of inhibiting this phenomenon. This stabilization counteracted hypoxia and has impaired the binding of PD1 to PD-L1 on endothelial cells in a melanoma and mammary carcinoma model, thereby reflecting the role of ITPP as an oxygen enhancer and vascular stabilizer in hypoxic tumors [20, 21].

### Targeting angiogenesis

#### Anti-VEGF therapy: bevacizumab and SU1498

Due to the explicit contribution of angiogenesis in the aggressiveness of GBM, bevacizumab, a monoclonal antibody targeting VEGF-A, has been approved by the FDA since 2004 for clinical use, and as a second-line treatment along with chemotherapy. Bevacizumab inhibits the binding of VEGF to its receptors, FLT-1 and KDR, on the surface of endothelial cells, resulting in the reduction of tumor vascularization and a subsequent reduction in tumor growth. Unfortunately, resistance towards this antibody has been reported, where GLUT1 and HIF-1α increased in two GBM cell lines reflecting an elevated glucose consumption [22]. Interestingly, a preclinical study showed that the response of GBM cell lines to bevacizumab treatment was dependent on the VEGF-A level of expression, where they were able to calculate the dose of antibody required to increase the response of GBM to this therapy [23]. Based on their findings, the authors suggested measuring VEGF-A levels in serum as a biomarker to identify the responsive patient population.

In a similar context, SU1498 is a selective inhibitor of VEGFR2, which accumulates the phosphorylated ERK1/2 in endothelial cells by blocking its kinase activity. After irradiation, the level of VEGF increases which might explain in part the radioresistance phenomenon observed in GBM towards conventional therapies. The addition of SU1498 to irradiation showed an enhanced radiosensitization of GBM cell lines and exhibited an additive effect on the apoptosis of A172 and U-251 MG GBM cell lines in vitro [24]. Similarly, the combination of SU1498 with TMZ sensitized GBM via the downregulation of Neuropilin-1 [25].

#### Integrin inhibition: cilengitide and Si306

Due to the role integrins play in the cell-cell and cell-stromal interactions, inhibiting integrins have been the focus in several therapeutic strategies targeting GBM. For instance, cilengitide (C_27_H_40_N_8_O_7_) is a pentapeptide that selectively inhibits the activation of αvβ3 and αvβ5 integrins that regulate different aspects of tumor survival including angiogenesis [26]. In GBM, preclinical studies have shown that the inhibition of integrins by cilengitide restricted the EGFRvIII/integrin β3 complex formation in hypoxic conditions, reduced tumor migration, and exhibited tumor regression and angiogenesis inhibition in orthotopic xenografts [27]. Interestingly, the nanoparticle encapsulation of cilengitide along with ultrasound-targeted microtubule destruction ameliorated the biodistribution of cilengitide and led to a buildup in the tumor site of a rat GBM model, in addition to increasing apoptosis and autophagy [28].

After the formation of EGFRvIII/integrin β3 complex under hypoxic conditions, the SRC proto-oncogene non-receptor tyrosine kinase (c-SRC) is activated leading to the upregulation of MMP-2 and -9 involved in cellular migration. Interestingly, Si306 is an antagonist of SRC, and has been shown to radiosensitize GBM cell lines via decreasing cell proliferation and clonogenic capacity, and increasing the number of γH2AX foci generation post-radiation [29]. In addition, other downstream signaling pathways activated by SRC were also reduced after Si306 treatment, including FAK and EGFR expression [30]. Finally, one important feature of Si306 is its ability to cross the BBB detected in mice brain post-intravenous injection [31].

#### Other molecules targeting angiogenesis

Other investigated anti-angiogenic molecules used to target GBM include cediranib, vandetanib [32], and galunisertib, with the latter being also a TGF-β inhibitor [33, 34]. In addition, celastrol which inhibits the PI3K/Akt/mTOR pathway, has been found to reduce the vasculogenic mimicry and angiogenesis in GBM [35]. Isolinderalactone, which suppresses the expression of VEGF, has been proven to reduce HIF-1α and HIF-2α protein expression along with the migration and capillary-like tube formation of HBMECs under hypoxia [36]. Moreover, isoliquiritigenin, a natural compound and member of the flavonoids, has been shown to prolong the growth of C6 glioma rats, normalized the vasculature, thus prolonging survival, and significantly increased the OS when combined with TMZ, through the downregulation of HIF-1α, FGF-2, VEGF, and TGF-β [37]. Finally, single-cell and spatial transcriptomic analyses of myeloid cells from GBM patients revealed the presence of a spatial association between polarized tumor-associated macrophages with a hypoxia signature, and destabilized microvessels in the perinecrotic regions of GBM, thus contributing to the hyperpermeable characteristic of GBM vasculature. Results showed that the inhibition of adrenomedullin, which is secreted by polarized hypoxia-activated TAMs might represent a new opportunity to normalize tumor vasculature [38].

### Inhibition of cellular oxygen consumption

#### Anti-parasitic drugs

Anti-parasitic drugs are used to target mitochondrial metabolism and reduce the oxygen consumption rate. For instance, atovaquone, an FDA-approved anti-malarial drug, targets the mitochondria via inhibiting the electron transport into complex III [39]. Studies have shown that formulated atovaquone effectively reduced the cellular viability of GBM cell lines in vitro via the inhibition of STAT3 [40]. Similarly, the FDA-approved drug doramectin, has been shown to inhibit the PI3K/AKT/mTOR pathway and induce autophagy [41]. Likewise, ivermectin, which has been included in clinical trials of COVID-19, induced apoptosis, and cell cycle arrest in GBM cell lines both in vitro and in vivo [42]. Interestingly, ivermectin interferes at the mitochondrial level by reducing the respiration, increasing the superoxide levels, and inhibiting the Akt/mTOR pathway to induce oxidative stress [43]. Other anti-parasitic drugs exit including mefloquine, chloroquine, and quinacrine among others, all of which are candidates for a potential clinical application in the treatment of GBM [39].

#### Metformin

The anti-diabetic agent metformin has been suggested as an important candidate in GBM preclinical studies targeting hypoxia due to its capability of modulating cellular metabolism and reducing the oxygen consumption rate. Cells with a TMZ-resistant phenotype such as T98, manifested an increased HIF-1α activity after treatment with TMZ, however, the addition of metformin to TMZ treatment was able to decrease the expression of HIF-1α in these cell lines [44]. In addition, since metformin reduces the oxygen consumption rate, more oxygen could be available for tumor cells, which was able to reverse the hypoxia-induced specific gene signature discovered in GBM cell lines [45]. Moreover, when administered alone, metformin was able to decrease cellular viability, increase apoptosis, inhibit cell migration and EMT, and increase the mitochondrial ROS production of three GBM cell lines in vitro, and most importantly, the concentration of TMZ used was reduced upon the addition of metformin [46]. Despite these promising results, one inconvenience of metformin administration is the associated lactate production and the increased risk of related acidosis. However, metformin is still considered an attractive strategy for future combinations with immunotherapy due to its low toxicity profile deduced from its clinical applications over the years [45].

### FLASH radiotherapy

FLASH radiotherapy (FLASH-RT), a new technique delivering ultra-high dose rate radiation, aims to reduce normal tissue toxicity while preserving an efficient tumor control to address the limitations of conventional RT [47]. Recently, FLASH-RT has been proposed as a promising approach to sensitize hypoxic tumors. A single fraction of 20 Gy (Oriatron/eRT6; mean dose rate >100 Gy/s) was delivered to a hypoxic subcutaneous mouse model of the U-87 GBM human cell line accomplished by a vascular clamp before and during the irradiation process. Interestingly, only FLASH-RT was able to maintain the tumor control efficacy under severe hypoxia, reflecting its ability to overcome the hypoxia-induce radioresistance, and hence offering a better treatment option to target tumor hypoxia while sparing normal tissues [48].

## GBM organoids as a preclinical model of hypoxia

Due to the intricate development of hypoxia in preclinical cell lines cultured in 2D systems, an increased attention towards the development of hypoxia in 3D models has been recognized over the past few years to ameliorate in vitro GBM hypoxia studies. However, despite extensive research on GBM organoids, few studies have tackled the employment of such models for the investigation of hypoxia. The focus of organoid models is the enrichment of the cancer stem cell subpopulation that is hypothesized for being responsible for the tumor-acquired resistance to treatments and the eventual recurrence. Yet, there exists a correlation between the maintenance and differentiation of the cancer stem/progenitor cells and the presence of hypoxia within the GBM bulk. For instance, researchers successfully generated patient-derived GBM organoids with stemness properties and detected a HIF-1α positive core using a bioreactor with optimized shear stress. GBM organoids also demonstrated transdifferentiation into different lineages [49]. Likewise, the GBM organoid model was used to isolate quiescent cells, which exhibited higher treatment resistance than the proliferative cells. Transcriptome analysis revealed a mesenchymal shift in this quiescent population promoted by hypoxia and TGF-β signaling [50]. Moreover, the presence of a hypoxic gradient was observed in patient-derived GBM organoids cultured under optimized conditions [51]. Noteworthy, by using GBM organoid model, the adaptation of cancer cells to harsh environment has been linked to the activation of the alternative RNA splicing events, such as asparagine endopeptidase (AEP) specifically cleaved DDX3X mediated by HIF-1α signaling [52]. Recently, cerebral organoids were cultured with patient-derived glioma stem cells (GSCs) to monitor tumor progression over an extended period. Interestingly, the development of chronic hypoxia and oxidative stress with time were linked with the expansion of GSCs via microenvironment remodeling through the secretion of pro-tumorigenic ligands such as FGF1 [53]. Therefore, the preclinical implication of such models represent a robust tool in GBM studies, considering the heterogeneity of GBM and the variations in its extracellular matrix components, such as the hypoxic gradients and their effect on GBM aggressiveness. Additionally, these models may serve as potential screening tools in studies that target hypoxia.

## Clinical strategies targeting hypoxia in GBM

### Reoxygenation

#### Hyperbaric oxygen therapy

Hyperbaric oxygen treatment (HBOT) involves high atmospheric pressures (>1 ATA) with 100% O_2_ to increase the oxygen tissue level, improve oxygen supply, and reduce inflammation and edema. In a phase II trial, 57 patients out of which 39 were high-grade gliomas (HGGs), were treated with daily conventional fractionated RT (60 Gy/30 fractions) 15 min post-HBOT with multi-agent CT in a multimodal approach. All patients completed the RT + HBOT protocol; however, 44 were not able to complete the designated CT courses. Long-term results showed that the median OS of the 39 patients with GBM was 17.2 months, while almost 48% of patients developed acute toxicities [54]. Moreover, results from another trial assessing the effect of IMRT boost after HBOT concurrent with TMZ-based CT were promising, with the median OS at 22.1 months. However, because several therapies were combined together, the improved OS and the acceptable tolerance were designated to HBOT only [55]. In addition, combining HBOT with re-irradiation using fractionated stereotactic RT (FSRT) in a small cohort of recurrent glioma patients showed that the median OS was 11 months for GBM patients. Although the dose was low, 22 Gy in 8 fractions, the authors reported 7 cases of re-operation, some of them for necrosis. However, some limitations accompany such approach including a long irradiation time, probably longer than the presence of O_2_ in the tumor post-HBOT in addition to the low doses delivered per fraction [56]. Finally, in a pilot study of patients with recurrent HGG who received daily fractions of 5 Gy for 3–5 consecutive days, delivered within 1 h post-HBOT, the median PFS and the OS were 5.2 months and 10.7 months, respectively, while limited neurotoxicities were observed. However, the authors were unable to fix the interval between HBOT and RT and used instead a range between 4 and 50 min [57]. Results of a systematic review showed that the addition of HBOT to RT is tolerated and may be beneficial in patients with HGG [58]. However, most of the analyzed trials failed to include the HBOT in a randomized manner among patients, while most studies were heterogeneous and lacked a sham method to assess the efficacy of HBOT, thus reducing its relevance [59]. Finally, the distance between facilities poses logistical challenges in coordinating HBOT and RT.

#### Carbogen and nicotinamide

Breathing carbogen before and during irradiation increases the dissolved oxygen in the plasma and reduced chronic hypoxia, while nicotinamide reduces acute hypoxia via increasing the uniformity of tumor blood perfusion and reducing intermittent blood vessels closure. The association of carbogen (95% O_2_ plus 5% CO_2_) and nicotinamide in preclinical studies, radiosensitized tumors after both single and fractionated irradiations. However, clinical studies on the association of carbogen-nicotinamide (CN) in GBM date back to the late 1990s, with no advantage on OS along with tolerance issues and difficult to resolve side effects. In a phase II study, 33 patients with an inoperable GBM, were treated with RT, CN, and intra-arterial cerebral CT compared with another group of 38 patients with no CN. No advantage was observed with the addition of CN, with nicotinamide tolerance issues leading to treatment termination for some patients [60]. Due to encountered toxicities and lack of interest, further development of CN therapy alongside RT has been halted.

#### Trans-sodium crocetinate

TSC, a synthetic carotenoid, has been studied in several clinical trials and is currently in a phase III clinical trial in GBM patients (NCT03393000) due to its role as an oxygen diffusion enhancer. In a prior GBM open, single-arm clinical trial, TSC was given with concomitant RT and TMZ 45 min before every RT session 3 days/week over 6 weeks based on phase-I safety assessment [61]. No dose-limiting toxicity had occurred post-injection of the 18 TSC sessions, leading to Phase II with 56 patients of which 36% who received the full dose of TSC were alive at 2 years versus 27% for RT and TMZ group. Serious adverse events (AEs) were reported in 12 patients but were not attributed to TSC. Fifty-two patients reported AEs in which 19% were TSC-associated [61]. Interestingly, biopsy-only GBM patients exhibited a similar survival outcome as resected patients when treated with TSC. Therefore, an open label phase III study will be initiated including GBM biopsy-only patients, in addition to the extension of TSC over the adjuvant TMZ period, which might improve the outcome of GBM. However, the clinical translation of TSC still faces some major issues including its solubility and short half-life. Therefore, a liposomal encapsulation with an active moiety of TSC has been developed (LEAF-4L6715) and the reported patient tolerability might represent a breakthrough in the development of these oxygen-enhancing carriers [62].

#### Dodecafluoropentane emulsion

Dodecafluoropentane emulsion (DDFPe) is a perfluorocarbon with a pentane base forming an emulsion of nanodroplets. DDFPe can carry large amounts of oxygen at temperatures >29 °C (boiling point), and due to their small particle size, they can reach hypoxic regions that are mostly not accessible by erythrocytes [63]. According to the premises of this emulsion as an oxygen therapeutic, a phase Ib/II dose-escalation study was initiated to assess the safety and efficacy of DDFPe (dose-escalation) in the treatment of newly diagnosed GBM patients along with the first-line radiochemotherapy in addition to breathing of carbogen (98% oxygen and 2% CO_2_) or 100% oxygen [64]. Results showed that the administration of DDFPe before every RT session was safe and tolerable; however, dose-escalation has been interrupted due to the development of symptomatic radionecrosis in few cases. Noteworthy, TOLD MRI confirmed the reoxygenation status and the reversal of hypoxia post-DDFPe in combination with other treatments. Even though the PFS and OS were increased as compared to previous controls, yet the sample size remains a limitation in this study (11 patients out of which two developed radionecrosis).

### Targeting angiogenesis

#### Avastin (bevacizumab)

Bevacizumab have been approved by the FDA for the treatment of recurrent GBM patients or in combination with the first-line treatments for primary tumors, due to the encouraging radiological response rates and the increased PFS rates [65, 66]. However, the clinical implication of bevacizumab is not widely adapted due to the controversial analysis of trial results. Despite the observed beneficial effects on the PFS, no positive impact was demonstrated on the OS outcome in some phase III randomized controlled trials [67–69]. In a phase II trial, combining bevacizumab with the NCIC/EORTC treatment protocol improved the PFS when compared to local and NCIC/EORTC cohorts, but not the OS, in addition to important grade 3–4 complications [70]. Conversely, another phase II trial incorporating irinotecan with bevacizumab in relapse treatment, showed an increase of the OS [71]. OS comparisons with the NCIC/EORTC trial were favorable which led to the initiation of phase III clinical trials such as the RTOG 0825 (637 patients), comparing the NCIC/EORTC protocol with bevacizumab versus placebo. The PFS improved but not the OS since the predefined criteria were not fully met, in addition to the presence of a higher cognitive degradation in the bevacizumab group, which might be linked to the exclusion of biopsy-only patients [68]. Similarly, in the AVAGlio trial, the PFS increased significantly but the OS did not improve [72]. Despite the improved QoL with the triple therapeutic approach, bevacizumab’s impact has been limited by increased side effects and complications [72]. A retrospective study demonstrated an improvement in the PFS and OS in patients undergoing RT with bevacizumab versus RT only. However, variable protocols for bevacizumab delivery during or after RT have complicated the interpretation [73]. Likewise, in a study focusing on combined chemotherapy, the PFS and OS improved in patients who received bevacizumab versus those that underwent RT or treated with variable chemotherapy drugs [74]. A targeted study on HGG with reduced treatment variability showed distinct relapse patterns in patients treated with 3D-RT or IMRT alongside bevacizumab. Notably, the planning target volume (PTV) was a prognostic factor for the PFS [75]. Moreover, bevacizumab improved the PFS in MGMT-negative patients, those with low-volume relapsed tumor, and those who received high irradiation dose [76]. In addition, bevacizumab with high irradiation dose improved the tumor control in patients treated for at least one relapse area [77]. In recurrent GBM, combining bevacizumab with re-irradiation showed safety, tolerability, and effectiveness, leading to an improved PFS and OS [78]. Concurrent irradiation and bevacizumab reduced the risk of radionecrosis after re-irradiation and the PFS increased when patients received first RT followed by bevacizumab during the new relapse [79, 80]. A phase I trial combining re-irradiation, bevacizumab, and pembrolizumab in patients with recurrent GBM, reported encouraging results on the PFS and OS, particularly in bevacizumab-naïve cohort at the time of re-irradiation [81]. Notably, the NRG Oncology/RTOG1205 phase II trial was the first randomized multi-institutional study to evaluate the safety and efficacy of concurrent bevacizumab with re-irradiation versus bevacizumab alone for recurrent GBM using modern RT techniques. Overall, the re-irradiation was safe and well tolerated, with improved PFS (6-month rate) in the treatment combination group, but no difference in the OS has been reported [82]. Notably, the total irradiation dose was limited to 35 Gy in 10 fractions using various techniques, except for the stereotactic irradiation despite its common use in retrospective studies. Finally, results from a retrospective study combining bevacizumab to chloroquine, an autophagy inhibitor, showed a synergistic effect with a significant increase in the OS at 36.5 months for patients who received radiochemotherapy, bevacizumab and chloroquine, versus 26.8 months in patients who received radiochemotherapy and bevacizumab. However, the number of patients included in this study was very small to draw a conclusive effect of such combination [83].

### Cilengitide

Encouraging results from phase II clinical trials led to the incorporation of cilengitide in combination with TMZ and RT in a randomized open-label phase III trial [84]. Patients were selected based on their MGMT promoter methylation, age (>18 years old), and GBM histological location (supratentorial). Cilengitide was administered up to 18 months with or without TMZ over 6 cycles. Although the addition of cilengitide did not cause further overall toxic effects, results from this multicenter randomized trial failed to show an improvement on the OS of newly diagnosed GBM patients, therefore impeding the integration of cilengitide in further studies on GBM.

Other anti-angiogenic molecules such as cediranib (45 mg) or vandetanib (100 mg/day) were also assessed in newly diagnosed GBM patients in combination with TMZ and RT Similar results were observed with both molecules with no significant effect of this combination on the OS and higher AEs linked to cediranib [85].

### Inhibition of cellular oxygen consumption

#### Metformin

Few clinical trials have been conducted on metformin in GBM. Recently, a prospective, randomized, multicenter phase II trial has assessed the safety and efficacy of combining metformin to low doses of TMZ in patients with recurrent GBM [86]. 32 patients in the experimental group received daily doses of metformin (2000 mg/mL) and low-dose TMZ (50 mg/m^2^). Metformin was safe and tolerable; however, no significant difference was detected between the median OS in experimental group versus TMZ group (log-rank test), and results were similar for disease control rate and the good response rate, with no significant difference between both groups. Finally, a higher frequency of MGMT methylation status has been detected in TMZ group when compared with the experimental group. Other clinical trials combining metformin with TMZ and RT are ongoing, with no published results so far.

### Hypoxia-activated prodrugs

#### Evofosfamide

Evofosfamide (TH302) is a second-generation hypoxia-activated prodrug with an ameliorated diffusion rate into hypoxic regions, due to its reduction into the cytotoxic and diffusive bromo-isophosphoramide mustard (Br-IPM). This strategy seems attractive to activate the nitroimidazole prodrugs, especially in hypoxic recurrent GBM cases post-treatments. Consequently, a single-arm phase II study assesses the combination of bevacizumab with TH302 in bevacizumab refractory GBM patients after proving that the combination of evofosfamide and bevacizumab was safe in a prior phase I trial [87]. Results showed no significant improvement on the PFS and OS of patients receiving the combination of evofosfamide. In a complementary study, the effect of such combination has been used to determine circulating metabolic markers of tumor hypoxia and to evaluate early response to treatment. Metabolites such as γ-aminobutyric acid, glutamic acid and D-glutamic acid were inversely correlated with tumor hypoxia observed with ^18^F-misoinidazole PET imaging [88]. This approach might be helpful in selecting potential responsive patients to evofosfamide and bevacizumab combination following bevacizumab treatment failure based on alterations in their hypoxia-induced metabolic profile.

## Limitations to the clinical translation of hypoxia-targeting protocols

**Fig. 1:**
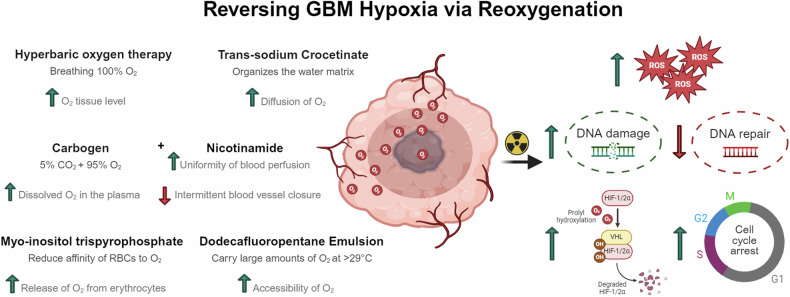
Tumor reoxygenation strategies and their respective biological outcome on GBM. The presence of oxygen upon treatment delivery, especially radiotherapy, increases the damage caused by such treatments on the DNA. In the presence of oxygen, more indirect DNA damage is generated due to the formation of ROS and the persistence of peroxyl adducts that are harder to repair, in addition to an increase in the cell cycle arrest, thus sensitizing GBM to conventional treatments. As the diffusion of oxygen increases through the tumor, other hypoxia-induced adaptations are reversed such as the increased degradation of HIF-1/2α in the presence of oxygen.

**Fig. 2:**
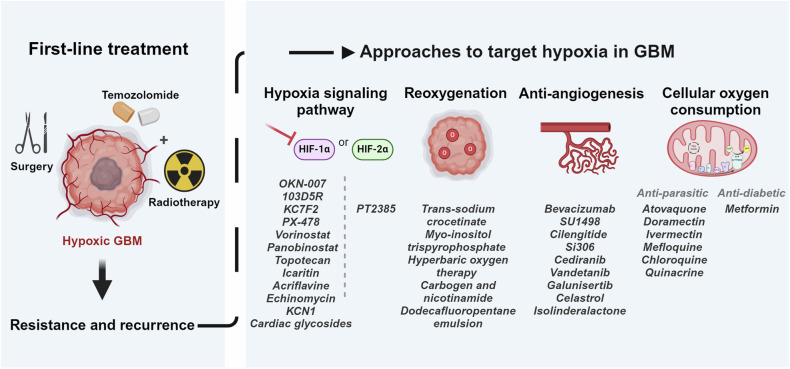
Preclinical and clinical approaches to target tumor hypoxia in GBM. The first-line therapy constitutes of a multimodal approach including surgery and chemoradiotherapy, yet GBM has been shown to exhibit resistance to such medical intervention. Although chronic or diffusion-limited hypoxia represents one of the pillars driving this aggressive behavior, it is overlooked in the diagnosis and treatment of GBM. Several preclinical and clinical approaches have been suggested as methods to reverse the effect of hypoxia on GBM response to conventional treatments. These include the inhibition of the hypoxia signaling pathway via HIFs, increasing the presence of oxygen within the tumor bulk or reoxygenation, targeting angiogenesis the main driver of tumor hypoxia, and reducing the cellular oxygen consumption via mitochondrial or cellular metabolism.

As discussed in the above sections, several preclinical and clinical approaches have been suggested to counteract hypoxia and sensitize GBM to conventional therapies. Strategies that target hypoxia include the inhibition of hypoxia signaling pathway via HIF-1α and HIF-2α inhibition, reoxygenation approaches to increase the presence of oxygen and reverse the effect of hypoxia (Fig. 1), inhibition of angiogenesis, which participates in maintaining tumor hypoxia, in addition to other strategies, which induce modifications on the oxygen metabolism and cellular respiration (Fig. 2). Although targeting hypoxia in GBM offers a promising perspective in the management of GBM resistance, yet some limitations accompany the proposed schemes (summarized in Table 2). For instance, acquired resistance to the used inhibitors represents the major pitfall of such approaches. Other observed phenomena include tolerance and toxicity issues such as grade 3–4 complications and radionecrosis. Although some of the hypoxia-targeted medications have successfully increased the PFS of GBM patients, yet none had a significant effect on improving the OS when administered alone or in combination with first-line treatments. Finally, some of the proposed strategies are not yet integrated into clinical trials and thus no further information on their safety or tolerability is known. On the other hand, there exist other proposals that might as well be considered as methods to target tumor hypoxia but were not discussed in this review. For instance, carbon ion therapy induces damage to the DNA regardless of the oxygen concentration, while dose-escalation deposit with IMRT or hypoxia-targeted dose painting, might be interesting approaches to target hypoxic sites within GBM.

**Table 2:** Preclinical and clinical strategies for targeting hypoxia in newly diagnosed and recurrent GBM and their limitations upon the clinical translation.

Strategies to target hypoxia in GBM	Molecules	Mode of action	Limitations
Hypoxia signaling pathways	OKN-007, 103D5R, KC7F2, Cardiac glycosides, PX-478, Vorinostat, Panobinostat, Topotecane, Icaritin, Acriflavine, Echinomycin, KCN1	Inhibit HIF-1α	Risks of unintended adverse side effects linked to the ubiquitous expression of HIF-1α in non-tumor tissue [8]
	PT2385	Inhibits HIF-2α	- PT2385, presented anti-tumoral activity in a phase I trial (*NCT02293980*) in patients with clear renal cell carcinoma, but no responses were demonstrated in a phase II (*NCT03216499*) trial with recurrent GBM patients - Limited activity in first recurrent GBM, acidic lesions were correlated with treatment duration *(NCT03216499)*
Reoxygenation	Trans-sodium crocetinate	Enhances oxygen diffusion by organizing the water matrix	Diffusion, stability, and bioavailability issues [62]
	Myo-inositol trispyrophosphate	Enhances oxygen release from RBCs and stabilizes tumor vascularization	No sufficient data and no clinical trials on GBM models
	Hyperbaric oxygen therapy	Elevates atmospheric pressure (>1 ATA) with 100% O_2_	No sham group was used to assess the efficacy of HBOT and the timespan between HBOT and RT seemed unreasoned [59]
	Carbogen and nicotinamide	- Increase dissolved oxygen - Increase the uniformity of tumor blood perfusion and reduce the intermittent closure of blood vessels	Tolerance issues and increased toxicities [60]
	Dodecafluoropentane emulsion	- Carries large amounts of oxygen - Enhances oxygen deposit	- Radionecrosis [64] - No sufficient data on OS in GBM
Anti-angiogenesis	Bevacizumab	Neutralizes the biological activity of VEGF-A	- Beneficial effects on the PFS [74] - No positive impact on the OS outcome [68, 72] - Grade 3–4 complications including cerebrovascular events and embolism [70]
	SU1498	Selectively inhibits VEGFR2	No clinical trials available on GBM
	Cilengitide	Selectively inhibits the activation of αvβ3 and αvβ5 integrins	Did not improve the OS of GBM patients upon combination with TMZ [84]
	Si306	Antagonizes c-SRC	No clinical trials available on GBM
	Cediranib, vandetanib	Inhibit VEGF receptor tyrosine kinase	Did not improve the PFS or OS of GBM patients upon combination with TMZ and RT [85]
	Galunisertib	Inhibits TGF-β	No clinical data results available on GBM
	Celastrol	Inhibits PI3K/Akt/mTOR pathway	No clinical trials available on GBM
	Isolinderalactone	Suppresses the expression of VEGF	No clinical trials available on GBM
Cellular oxygen consumption	Atovaquone, Doramectin, Ivermectin, Mefloquine, Chloroquine, and Quinacrine	- Anti-parasitic drugs - Inhibit the mitochondrial respiration	Only chloroquine have been assessed in GBM clinical trials with no published results so far
	Metformin	-Anti-diabetic drug (type II diabetes) - Reprograms oxygen metabolism	Did not improve the OS of patients upon combination with low-dose TMZ in refractory and recurrent GBM [86]

## Conclusion

Tumor hypoxia has been identified as one of the main drivers of GBM aggressive behavior. The presence of hypoxia within the tumor bulk and microenvironment leads to the activation of numerous genes and consequent biological pathways involved in angiogenesis, metabolic shift, autophagy, mesenchymal shift, and immunosuppression among others, thus increasing the resistance of GBM to the first-line treatments even after a multimodal therapeutic approach. Hence, targeting hypoxia might reverse its drastic effects on GBM outcome. To that end, several approaches have been explored in both preclinical and clinical settings including the inhibition of hypoxia signaling pathway, tumor reoxygenation, inhibition of angiogenesis, and the inhibition of the cellular oxygen consumption. In addition, due to the recency of some strategies, no clinical data is available so far, such as the use of UHDR radiation to target hypoxia (FLASH-RT). Also, due to the complexity of hypoxia development in the preclinical setting, more focus have been directed towards GBM organoids as models for hypoxia development and might represent a robust tool in preclinical studies that aim to target hypoxia. On the other hand, hypoxia is still overlooked in the clinical assessment of GBM. Consequently, the integration of hypoxia in the diagnosis of GBM might offer a new insight on better addressing one of the main drivers of this tumor. In addition, hypoxia-induced transcriptomic and proteomic analysis, as well as advanced MRI and PET imaging of hypoxia, should be integrated in routine clinical practice to offer a personalized multimodal approach that might improve therapy outcome in GBM patients, while maintaining an acceptable QoL. Finally, patient selection should be performed with great caution to address those that might benefit from the proposed approaches based on their hypoxia signature.

## References

[1] Stupp R, Mason WP, van den Bent MJ, Weller M, Fisher B, Taphoorn MJ (2005). Radiotherapy plus concomitant and adjuvant temozolomide for glioblastoma. N Engl J Med.

[2] Stupp R, Hegi ME, Mason WP, van den Bent MJ, Taphoorn MJ, Janzer RC (2009). Effects of radiotherapy with concomitant and adjuvant temozolomide versus radiotherapy alone on survival in glioblastoma in a randomised phase III study: 5-year analysis of the EORTC-NCIC trial. Lancet Oncol.

[3] Birzu C, French P, Caccese M, Cerretti G, Idbaih A, Zagonel V (2020). Recurrent glioblastoma: from molecular landscape to new treatment perspectives. Cancers. Cancers.

[4] Vymazal J, Kazda T, Novak T, Slanina P, Sroubek J, Klener J (2022). Eighteen years’ experience with tumor treating fields in the treatment of newly diagnosed glioblastoma. Front Oncol.

[5] Stupp R, Taillibert S, Kanner A, Read W, Steinberg D, Lhermitte B (2017). Effect of tumor-treating fields plus maintenance temozolomide vs maintenance temozolomide alone on survival in patients with glioblastoma: a randomized clinical trial. JAMA.

[6] Xu R, Wang F, Yang H, Wang Z. Action sites and clinical application of HIF-1alpha inhibitors. Molecules. 2022;27:3426. 10.3390/molecules27113426 PMC9182161 35684364

[7] Albadari N, Deng S, Li W (2019). The transcriptional factors HIF-1 and HIF-2 and their novel inhibitors in cancer therapy. Expert Opin Drug Discov.

[8] Renfrow JJ, Soike MH, West JL, Ramkissoon SH, Metheny-Barlow L, Mott RT (2020). Attenuating hypoxia driven malignant behavior in glioblastoma with a novel hypoxia-inducible factor 2 alpha inhibitor. Sci Rep.

[9] Wallace EM, Rizzi JP, Han G, Wehn PM, Cao Z, Du X (2016). A small-molecule antagonist of HIF2alpha is efficacious in preclinical models of renal cell carcinoma. Cancer Res.

[10] Gainer JL (2008). Trans-sodium crocetinate for treating hypoxia/ischemia. Expert Opin Investig Drugs.

[11] Colapietro A, Mancini A, Vitale F, Martellucci S, Angelucci A, Llorens S (2020). Crocetin extracted from saffron shows antitumor effects in models of human glioblastoma. Int J Mol Sci.

[12] Shah HM, Jain AS, Joshi SV, Kharkar PS (2021). Crocetin and related oxygen diffusion-enhancing compounds: review of chemical synthesis, pharmacology, clinical development, and novel therapeutic applications. Drug Dev Res.

[13] Sheehan J, Ionescu A, Pouratian N, Hamilton DK, Schlesinger D, Oskouian RJ (2008). Use of trans sodium crocetinate for sensitizing glioblastoma multiforme to radiation: laboratory investigation. J Neurosurg.

[14] Wong KH, Xie Y, Huang X, Kadota K, Yao XS, Yu Y (2020). Delivering crocetin across the blood-brain barrier by using gamma-cyclodextrin to treat Alzheimer’s disease. Sci Rep.

[15] Duarte CD, Greferath R, Nicolau C, Lehn JM (2010). myo-Inositol trispyrophosphate: a novel allosteric effector of hemoglobin with high permeation selectivity across the red blood cell plasma membrane. Chembiochem.

[16] Grgic I, Tschanz F, Borgeaud N, Gupta A, Clavien PA, Guckenberger M (2021). Tumor oxygenation by myo-inositol trispyrophosphate enhances radiation response. Int J Radiat Oncol Biol Phys.

[17] Tran LB, Cao-Pham TT, Jordan BF, Deschoemaeker S, Heyerick A, Gallez B (2019). Impact of myo-inositol trispyrophosphate (ITPP) on tumour oxygenation and response to irradiation in rodent tumour models. J Cell Mol Med.

[18] Fornvik K, Zolfaghari S, Salford LG, Redebrandt HN (2016). ITPP treatment of RG2 glioblastoma in a rat model. Anticancer Res.

[19] Iyengar S, Schwartz D (2017). Failure of inositol trispyrophosphate to enhance highly effective radiotherapy of GL261 glioblastoma in mice. Anticancer Res.

[20] El Hafny-Rahbi B, Brodaczewska K, Collet G, Majewska A, Klimkiewicz K, Delalande A (2021). Tumour angiogenesis normalized by myo-inositol trispyrophosphate alleviates hypoxia in the microenvironment and promotes antitumor immune response. J Cell Mol Med.

[21] Grzymajlo K, El Hafny-Rahbi B, Kieda C (2023). Tumour suppressor PTEN activity is differentially inducible by myo-inositol phosphates. J Cell Mol Med.

[22] Miranda-Goncalves V, Cardoso-Carneiro D, Valbom I, Cury FP, Silva VA, Granja S (2017). Metabolic alterations underlying Bevacizumab therapy in glioblastoma cells. Oncotarget.

[23] Garcia-Romero N, Palacin-Aliana I, Madurga R, Carrion-Navarro J, Esteban-Rubio S, Jimenez B (2020). Bevacizumab dose adjustment to improve clinical outcomes of glioblastoma. BMC Med.

[24] Knizetova P, Ehrmann J, Hlobilkova A, Vancova I, Kalita O, Kolar Z (2008). Autocrine regulation of glioblastoma cell cycle progression, viability and radioresistance through the VEGF-VEGFR2 (KDR) interplay. Cell Cycle.

[25] Lee J, Kim E, Ryu SW, Choi C, Choi K (2016). Combined inhibition of vascular endothelial growth factor receptor signaling with temozolomide enhances cytotoxicity against human glioblastoma cells via downregulation of Neuropilin-1. J Neurooncol.

[26] Reardon DA, Neyns B, Weller M, Tonn JC, Nabors LB, Stupp R (2011). Cilengitide: an RGD pentapeptide alphanubeta3 and alphanubeta5 integrin inhibitor in development for glioblastoma and other malignancies. Future Oncol.

[27] Liu Z, Han L, Dong Y, Tan Y, Li Y, Zhao M (2016). EGFRvIII/integrin beta3 interaction in hypoxic and vitronectinenriching microenvironment promote GBM progression and metastasis. Oncotarget.

[28] Zhao YZ, Lin Q, Wong HL, Shen XT, Yang W, Xu HL (2016). Glioma-targeted therapy using Cilengitide nanoparticles combined with UTMD enhanced delivery. J Control Release.

[29] Torrisi F, Minafra L, Cammarata FP, Savoca G, Calvaruso M, Vicario N (2020). SRC tyrosine kinase inhibitor and X-rays combined effect on glioblastoma cell lines. Int J Mol Sci.

[30] Nesovic M, Divac Rankov A, Podolski-Renic A, Nikolic I, Tasic G, Mancini A, et al. Src inhibitors pyrazolo[3,4-d]pyrimidines, Si306 and Pro-Si306, inhibit focal adhesion kinase and suppress human glioblastoma invasion in vitro and in vivo. Cancers. 2020;12:1570. 10.3390/cancers12061570 PMC7352231 32545852

[31] Fallacara AL, Zamperini C, Podolski-Renic A, Dinic J, Stankovic T, Stepanovic M, et al. A new strategy for glioblastoma treatment: in vitro and in vivo preclinical characterization of Si306, a pyrazolo[3,4-d]pyrimidine dual Src/P-glycoprotein inhibitor. Cancers. 2019;11:848. 10.3390/cancers11060848 PMC6628362 31248184

[32] Pham K, Luo D, Siemann DW, Law BK, Reynolds BA, Hothi P (2015). VEGFR inhibitors upregulate CXCR4 in VEGF receptor-expressing glioblastoma in a TGFbetaR signaling-dependent manner. Cancer Lett.

[33] Hadizadeh M, AminJafari A, Parvizpour S, Ghasemi S (2022). Novel targets to overcome antiangiogenesis therapy resistance in glioblastoma multiforme: Systems biology approach and suggestion of therapy by galunisertib. Cell Biol Int.

[34] Liu CC, Wu CL, Lin MX, Sze CI, Gean PW (2021). Disulfiram sensitizes a therapeutic-resistant glioblastoma to the TGF-beta receptor inhibitor. Int J Mol Sci.

[35] Zhu Y, Liu X, Zhao P, Zhao H, Gao W, Wang L (2020). Celastrol suppresses glioma vasculogenic mimicry formation and angiogenesis by blocking the PI3K/Akt/mTOR signaling pathway. Front Pharm.

[36] Park JH, Kim MJ, Kim WJ, Kwon KD, Ha KT, Choi BT (2020). Isolinderalactone suppresses human glioblastoma growth and angiogenic activity in 3D microfluidic chip and in vivo mouse models. Cancer Lett.

[37] Wang C, Chen Y, Wang Y, Liu X, Liu Y, Li Y (2019). Inhibition of COX-2, mPGES-1 and CYP4A by isoliquiritigenin blocks the angiogenic Akt signaling in glioma through ceRNA effect of miR-194-5p and lncRNA NEAT1. J Exp Clin Cancer Res.

[38] Wang W, Li T, Cheng Y, Li F, Qi S, Mao M (2024). Identification of hypoxic macrophages in glioblastoma with therapeutic potential for vasculature normalization. Cancer Cell.

[39] Mudassar F, Shen H, O’Neill G, Hau E (2020). Targeting tumor hypoxia and mitochondrial metabolism with anti-parasitic drugs to improve radiation response in high-grade gliomas. J Exp Clin Cancer Res.

[40] Takabe H, Warnken ZN, Zhang Y, Davis DA, Smyth HDC, Kuhn JG, et al. A repurposed drug for brain cancer: enhanced atovaquone amorphous solid dispersion by combining a spontaneously emulsifying component with a polymer carrier. Pharmaceutics. 2018;10:60. 10.3390/pharmaceutics10020060 PMC6027483 29783757

[41] Chen C, Liang H, Qin R, Li X, Wang L, Du S, et al. Doramectin inhibits glioblastoma cell survival via regulation of autophagy in vitro and in vivo. Int J Oncol. 2022;60:29. 10.3892/ijo.2022.5319 PMC8857930 35137919

[42] Song D, Liang H, Qu B, Li Y, Liu J, Zhang Y (2019). Ivermectin inhibits the growth of glioma cells by inducing cell cycle arrest and apoptosis in vitro and in vivo. J Cell Biochem.

[43] Liu Y, Fang S, Sun Q, Liu B (2016). Anthelmintic drug ivermectin inhibits angiogenesis, growth and survival of glioblastoma through inducing mitochondrial dysfunction and oxidative stress. Biochem Biophys Res Commun.

[44] Lo Dico A, Valtorta S, Ottobrini L, Moresco RM (2019). Role of metformin and AKT axis modulation in the reversion of hypoxia induced TMZ-resistance in glioma cells. Front Oncol.

[45] Calvo Tardon M, Marinari E, Migliorini D, Bes V, Tankov S, Charrier E (2020). An experimentally defined hypoxia gene signature in glioblastoma and its modulation by metformin. Biology.

[46] Feng SW, Chang PC, Chen HY, Hueng DY, Li YF, Huang SM. Exploring the mechanism of adjuvant treatment of glioblastoma using temozolomide and metformin. Int J Mol Sci. 2022;23:8171. 10.3390/ijms23158171 PMC9330793 35897747

[47] Friedl AA, Prise KM, Butterworth KT, Montay-Gruel P, Favaudon V (2022). Radiobiology of the FLASH effect. Med Phys.

[48] Leavitt RJ, Almeida A, Grilj V, Montay-Gruel P, Godfroid C, Petit B, et al. Acute hypoxia does not alter tumor sensitivity to FLASH radiation therapy. Int J Radiat Oncol Biol Phys. 2024;S0360-3016:00320-1. 10.1016/j.ijrobp.2024.02.015 38387809

[49] Park S, Avera AD, Kim Y (2022). Biomanufacturing of glioblastoma organoids exhibiting hierarchical and spatially organized tumor microenvironment via transdifferentiation. Biotechnol Bioeng.

[50] Tejero R, Huang Y, Katsyv I, Kluge M, Lin JY, Tome-Garcia J (2019). Gene signatures of quiescent glioblastoma cells reveal mesenchymal shift and interactions with niche microenvironment. EBioMedicine.

[51] Jacob F, Salinas RD, Zhang DY, Nguyen PTT, Schnoll JG, Wong SZH (2020). A patient-derived glioblastoma organoid model and biobank recapitulates inter- and intra-tumoral heterogeneity. Cell.

[52] Zhang W, Cao L, Yang J, Zhang S, Zhao J, Shi Z (2023). AEP-cleaved DDX3X induces alternative RNA splicing events to mediate cancer cell adaptation in harsh microenvironments. J Clin Invest.

[53] Nicholson JG, Cirigliano S, Singhania R, Haywood C, Shahidi Dadras M, Yoshimura M (2024). Chronic hypoxia remodels the tumor microenvironment to support glioma stem cell growth. Acta Neuropathol Commun.

[54] Ogawa K, Ishiuchi S, Inoue O, Yoshii Y, Saito A, Watanabe T (2012). Phase II trial of radiotherapy after hyperbaric oxygenation with multiagent chemotherapy (procarbazine, nimustine, and vincristine) for high-grade gliomas: long-term results. Int J Radiat Oncol Biol Phys.

[55] Yahara K, Ohguri T, Udono H, Yamamoto J, Tomura K, Onoda T (2017). Radiotherapy using IMRT boosts after hyperbaric oxygen therapy with chemotherapy for glioblastoma. J Radiat Res.

[56] Kohshi K, Yamamoto H, Nakahara A, Katoh T, Takagi M (2007). Fractionated stereotactic radiotherapy using gamma unit after hyperbaric oxygenation on recurrent high-grade gliomas. J Neurooncol.

[57] Arpa D, Parisi E, Ghigi G, Cortesi A, Longobardi P, Cenni P (2021). Role of hyperbaric oxygenation plus hypofractionated stereotactic radiotherapy in recurrent high-grade glioma. Front Oncol.

[58] Chen JR, Xu HZ, Ding JB, Qin ZY (2015). Radiotherapy after hyperbaric oxygenation in malignant gliomas. Curr Med Res Opin.

[59] Lansdorp CA, van Hulst RA (2018). Double-blind trials in hyperbaric medicine: a narrative review on past experiences and considerations in designing sham hyperbaric treatment. Clin Trials.

[60] Simon JM, Noel G, Chiras J, Hoang-Xuan K, Delattre JY, Baillet F (2003). Radiotherapy and chemotherapy with or without carbogen and nicotinamide in inoperable biopsy-proven glioblastoma multiforme. Radiother Oncol.

[61] Gainer JL, Sheehan JP, Larner JM, Jones DR (2017). Trans sodium crocetinate with temozolomide and radiation therapy for glioblastoma multiforme. J Neurosurg.

[62] Mertes PM, Collange O, Coliat P, Banerjee M, Diringer MC, Roche A (2021). Liposomal encapsulation of trans-crocetin enhances oxygenation in patients with COVID-19-related ARDS receiving mechanical ventilation. J Control Release.

[63] Woods SD, Skinner RD, Ricca AM, Brown AT, Lowery JD, Borrelli MJ (2013). Progress in dodecafluoropentane emulsion as a neuroprotective agent in a rabbit stroke model. Mol Neurobiol.

[64] Lickliter JD, Ruben J, Kichenadasse G, Jennens R, Gzell C, Mason RP (2023). Dodecafluoropentane emulsion as a radiosensitizer in glioblastoma multiforme. Cancer Res Commun.

[65] Cohen MH, Shen YL, Keegan P, Pazdur R (2009). FDA drug approval summary: bevacizumab (Avastin) as treatment of recurrent glioblastoma multiforme. Oncologist.

[66] Zhang G, Huang S, Wang Z (2012). A meta-analysis of bevacizumab alone and in combination with irinotecan in the treatment of patients with recurrent glioblastoma multiforme. J Clin Neurosci.

[67] Gilbert MR, Sulman EP, Mehta MP (2014). Bevacizumab for newly diagnosed glioblastoma. N Engl J Med.

[68] Gilbert MR, Dignam JJ, Armstrong TS, Wefel JS, Blumenthal DT, Vogelbaum MA (2014). A randomized trial of bevacizumab for newly diagnosed glioblastoma. N Engl J Med.

[69] Wick W, Gorlia T, Bendszus M, Taphoorn M, Sahm F, Harting I (2017). Lomustine and bevacizumab in progressive glioblastoma. N Engl J Med.

[70] Lai A, Tran A, Nghiemphu PL, Pope WB, Solis OE, Selch M (2011). Phase II study of bevacizumab plus temozolomide during and after radiation therapy for patients with newly diagnosed glioblastoma multiforme. J Clin Oncol.

[71] Vredenburgh JJ, Desjardins A, Reardon DA, Peters KB, Herndon JE, Marcello J (2011). The addition of bevacizumab to standard radiation therapy and temozolomide followed by bevacizumab, temozolomide, and irinotecan for newly diagnosed glioblastoma. Clin Cancer Res.

[72] Chinot OL, Wick W, Cloughesy T (2014). Bevacizumab for newly diagnosed glioblastoma. N Engl J Med.

[73] Cuneo KC, Vredenburgh JJ, Sampson JH, Reardon DA, Desjardins A, Peters KB (2012). Safety and efficacy of stereotactic radiosurgery and adjuvant bevacizumab in patients with recurrent malignant gliomas. Int J Radiat Oncol Biol Phys.

[74] Niyazi M, Ganswindt U, Schwarz SB, Kreth FW, Tonn JC, Geisler J (2012). Irradiation and bevacizumab in high-grade glioma retreatment settings. Int J Radiat Oncol Biol Phys.

[75] Niyazi M, Jansen NL, Rottler M, Ganswindt U, Belka C (2014). Recurrence pattern analysis after re-irradiation with bevacizumab in recurrent malignant glioma patients. Radiat Oncol.

[76] Flieger M, Ganswindt U, Schwarz SB, Kreth FW, Tonn JC, la Fougere C (2014). Re-irradiation and bevacizumab in recurrent high-grade glioma: an effective treatment option. J Neurooncol.

[77] Antoni D, Jastaniah Z, Haoming QC, Gaultier C, Ahle G, Couchot J (2016). Patterns of relapse in patients with high grade glioma receiving combined treatments including stereotactic re-irradiation for a first relapse. Cancer Radiother.

[78] She L, Su L, Liu C (2022). Bevacizumab combined with re-irradiation in recurrent glioblastoma. Front Oncol.

[79] Youland RS, Lee JY, Kreofsky CR, Brown PD, Uhm JH, Laack NN (2018). Modern reirradiation for recurrent gliomas can safely delay tumor progression. Neurooncol Pr.

[80] Morris SL, Zhu P, Rao M, Martir M, Zhu JJ, Hsu S (2019). Gamma knife stereotactic radiosurgery in combination with bevacizumab for recurrent glioblastoma. World Neurosurg.

[81] Sahebjam S, Forsyth PA, Tran ND, Arrington JA, Macaulay R, Etame AB (2021). Hypofractionated stereotactic re-irradiation with pembrolizumab and bevacizumab in patients with recurrent high-grade gliomas: results from a phase I study. Neuro Oncol.

[82] Tsien CI, Pugh SL, Dicker AP, Raizer JJ, Matuszak MM, Lallana EC (2023). NRG oncology/RTOG1205: a randomized phase II trial of concurrent bevacizumab and reirradiation versus bevacizumab alone as treatment for recurrent glioblastoma. J Clin Oncol.

[83] Witte HM, Riecke A, Steinestel K, Schulz C, Kuchler J, Gebauer N (2024). The addition of chloroquine and bevacizumab to standard radiochemotherapy for recurrent glioblastoma multiforme. Br J Neurosurg.

[84] Stupp R, Hegi ME, Gorlia T, Erridge SC, Perry J, Hong YK (2014). Cilengitide combined with standard treatment for patients with newly diagnosed glioblastoma with methylated MGMT promoter (CENTRIC EORTC 26071-22072 study): a multicentre, randomised, open-label, phase 3 trial. Lancet Oncol.

[85] Batchelor TT, Won M, Chakravarti A, Hadjipanayis CG, Shi W, Ashby LS (2023). NRG/RTOG 0837: Randomized, phase II, double-blind, placebo-controlled trial of chemoradiation with or without cediranib in newly diagnosed glioblastoma. Neurooncol Adv.

[86] Yoon WS, Chang JH, Kim JH, Kim YJ, Jung TY, Yoo H (2023). Efficacy and safety of metformin plus low-dose temozolomide in patients with recurrent or refractory glioblastoma: a randomized, prospective, multicenter, double-blind, controlled, phase 2 trial (KNOG-1501 study). Discov Oncol.

[87] Brenner AJ, Floyd J, Fichtel L, Michalek J, Kanakia KP, Huang S (2021). Phase 2 trial of hypoxia activated evofosfamide (TH302) for treatment of recurrent bevacizumab-refractory glioblastoma. Sci Rep.

[88] Lodi A, Pandey R, Chiou J, Bhattacharya A, Huang S, Pan X (2022). Circulating metabolites associated with tumor hypoxia and early response to treatment in bevacizumab-refractory glioblastoma after combined bevacizumab and evofosfamide. Front Oncol.

[89] Towner RA, Saunders D, Lerner M, Silasi Mansat R, Yuan T, Barber D (2021). Temporary opening of the blood-brain barrier with the nitrone compound OKN-007. Am J Nucl Med Mol Imaging.

[90] Towner RA, Smith N, Saunders D, Brown CA, Cai X, Ziegler J (2019). OKN-007 Increases temozolomide (TMZ) Sensitivity and Suppresses TMZ-Resistant Glioblastoma (GBM) Tumor Growth. Transl Oncol.

[91] Tan C, de Noronha RG, Roecker AJ, Pyrzynska B, Khwaja F, Zhang Z (2005). Identification of a novel small-molecule inhibitor of the hypoxia-inducible factor 1 pathway. Cancer Res.

[92] Abbaszade Z, Bagca BG, Avci CB (2021). Molecular biological investigation of temozolomide and KC7F2 combination in U87MG glioma cell line. Gene.

[93] Berges R, Denicolai E, Tchoghandjian A, Baeza-Kallee N, Honore S, Figarella-Branger D (2018). Proscillaridin A exerts anti-tumor effects through GSK3beta activation and alteration of microtubule dynamics in glioblastoma. Cell Death Dis.

[94] Lan YL, Chen C, Wang X, Lou JC, Xing JS, Zou S (2020). Gamabufotalin induces a negative feedback loop connecting ATP1A3 expression and the AQP4 pathway to promote temozolomide sensitivity in glioblastoma cells by targeting the amino acid Thr794. Cell Prolif.

[95] Terzioglu-Usak S, Nalli A, Elibol B, Ozek E, Hatiboglu MA (2020). Anvirzel(TM)regulates cell death through inhibiting GSK-3 activity in human U87 glioma cells. Neurol Res.

[96] Ding XC, Wang LL, Zhang XD, Xu JL, Li PF, Liang H (2021). The relationship between expression of PD-L1 and HIF-1alpha in glioma cells under hypoxia. J Hematol Oncol.

[97] Perez T, Berges R, Maccario H, Oddoux S, Honore S (2021). Low concentrations of vorinostat decrease EB1 expression in GBM cells and affect microtubule dynamics, cell survival and migration. Oncotarget.

[98] Rampazzo E, Manfreda L, Bresolin S, Cani A, Mariotto E, Bortolozzi R, et al. Histone deacetylase inhibitors impair glioblastoma cell motility and proliferation. Cancers. 2022;14:1897. 10.3390/cancers14081897 PMC9027190 35454804

[99] Nguyen TTT, Zhang Y, Shang E, Shu C, Torrini C, Zhao J (2020). HDAC inhibitors elicit metabolic reprogramming by targeting super-enhancers in glioblastoma models. J Clin Invest.

[100] Pratap UP, Sareddy GR, Liu Z, Venkata PP, Liu J, Tang W (2021). Histone deacetylase inhibitors enhance estrogen receptor beta expression and augment agonist-mediated tumor suppression in glioblastoma. Neurooncol Adv.

[101] Bernstock JD, Ye D, Gessler FA, Lee YJ, Peruzzotti-Jametti L, Baumgarten P (2017). Topotecan is a potent inhibitor of SUMOylation in glioblastoma multiforme and alters both cellular replication and metabolic programming. Sci Rep.

[102] Mo ZT, Li WN, Zhai YR, Gao SY (2017). The effects of icariin on the expression of HIF-1alpha, HSP-60 and HSP-70 in PC12 cells suffered from oxygen-glucose deprivation-induced injury. Pharm Biol.

[103] Li H, Liang Q, Wang L (2019). Icaritin inhibits glioblastoma cell viability and glycolysis by blocking the IL-6/Stat3 pathway. J Cell Biochem.

[104] Xu B, Jiang C, Han H, Liu H, Tang M, Liu L (2015). Icaritin inhibits the invasion and epithelial-to-mesenchymal transition of glioblastoma cells by targeting EMMPRIN via PTEN/AKt/HIF-1alpha signalling. Clin Exp Pharm Physiol.

[105] Mangraviti A, Raghavan T, Volpin F, Skuli N, Gullotti D, Zhou J (2017). HIF-1alpha-targeting acriflavine provides long term survival and radiological tumor response in brain cancer therapy. Sci Rep.

[106] Ma S, Wang F, Dong J, Wang N, Tao S, Du J (2022). Inhibition of hypoxia-inducible factor 1 by acriflavine renders glioblastoma sensitive for photodynamic therapy. J Photochem Photobio B.

[107] Peng G, Wang Y, Ge P, Bailey C, Zhang P, Zhang D (2021). The HIF1alpha-PDGFD-PDGFRalpha axis controls glioblastoma growth at normoxia/mild-hypoxia and confers sensitivity to targeted therapy by echinomycin. J Exp Clin Cancer Res.

[108] Yin S, Kaluz S, Devi NS, Jabbar AA, de Noronha RG, Mun J (2012). Arylsulfonamide KCN1 inhibits in vivo glioma growth and interferes with HIF signaling by disrupting HIF-1alpha interaction with cofactors p300/CBP. Clin Cancer Res.

